# Attitudes of Healthcare Personnel towards Vaccinations before and during the COVID-19 Pandemic

**DOI:** 10.3390/ijerph18052703

**Published:** 2021-03-08

**Authors:** Caterina Ledda, Claudio Costantino, Mario Cuccia, Helena C. Maltezou, Venerando Rapisarda

**Affiliations:** 1 Occupational Medicine, Department of Clinical and Experimental Medicine, University of Catania, Via Santa Sofia, 87 Edificio B Piano 0, 95123 Catania, Italy; vrapisarda@unict.it; 2 Department of Health Promotion Sciences, Maternal and Infant Care, Internal Medicine and Excellence Specialties, University of Palermo, Via del Vespro 129, 90127 Palermo, Italy; claudio.costantino01@unipa.it; 3 Epidemiology and Prevention Unit, Provincial Health Authority of Catania, Via Tevere, San Gregorio di Catania, 95027 Catania, Italy; mario.cuccia@aspct.it; 4 Directorate of Research, Studies and Documentation, National Public Health Organization, 3-5 Agrafon Street, 11523 Athens, Greece; helen-maltezou@ath.forthnet.gr

**Keywords:** SARS-CoV-2, COVID-19, healthcare personnel, occupational risk, vaccination, knowledge, attitudes, Italy, vaccine hesitancy

## Abstract

Vaccines constitute highly effective tools for controlling and eliminating vaccine-preventable diseases (VPDs) and are assessed to avert between two to three million deaths per year globally. Healthcare personnel (HCP) constitute a priority group for several vaccinations. However, studies indicate significant rates of vaccine hesitancy among them and, therefore, of acceptance of vaccination recommendations. This cross-sectional study was conducted in a university hospital in Southern Italy to assess the knowledge and attitudes of HCP about VPDs before and during the COVID-19 pandemic, estimate their intention to get vaccinated against COVID-19, and search for determinants that may influence their choice. A self-administered questionnaire was used. HCP improved their knowledge about VPDs and were more favorable to vaccinations in September–December 2020 compared to January–December 2019. Overall, 75% of respondents would get a COVID-19 vaccine. Our findings indicate a potential role of the ongoing COVID-19 pandemic on Italian HCP’s knowledge and attitudes towards vaccines.

## 1. Introduction

Vaccines constitute highly effective tools for controlling and eliminating vaccine-preventable diseases (VPDs) and are assessed to avert between two to three million deaths per year [1,2,3]. With prominently-defined target groups, they are among the most cost-effective public health investments. Evidence-based policies have given consent for them to be available to inform the most hard-to-reach and susceptible people [1,2,3]. Vaccination programs concern all age groups (from infants to the elderly), but they are frequently targeted to specific groups, such as pregnant women, travelers, and people with comorbidities [4,5,6,7].

One group intended for priority vaccination is healthcare personnel (HCP). Studies have shown significant gaps in knowledge towards VPDs and vaccines among HCP and, therefore, vaccination uptake rates among them [8,9]. This lack of HCP compliance towards vaccinations contributes to vaccine hesitancy, which detracts from vaccination programs’ effectiveness [10]. Vaccine hesitancy is defined as disinclination or refusal to be vaccinated against VPDs despite the availability of vaccines, and was recognized by the World Health Organization as one of the top 10 global health threats of 2019 [11,12,13]. Outbreaks due to VPDs continue to occur in various healthcare settings globally, threatening patients, HCP, and healthcare services [14,15,16,17,18,19,20,21]. Indeed, compliance with recommendations for vaccination among HCP is often suboptimal [22,23,24,25,26,27,28,29,30]. Consequently, to sustain vaccine-acquired herd immunity and vaccination policies, the immunization coverage of the HCP must reach the fixed threshold rates [31]. Understanding HCP knowledge and attitudes towards VPDs vaccination is essential to understand these issues and consent to recommend an opposite approach to advance immunization coverage rates [32,33]. Moreover, the newly developed vaccines against COVID-19 could be influenced by vaccine hesitancy among HCP [34].

Vaccine administration against COVID-19 has been underway for a couple of months in China and Russia, while the United Kingdom and the United States started their vaccination campaigns in early December 2020. In the European Union, the symbolic “Vaccine Day” was held on 27 December 2020 [35]. To date, there are scarce studies on HCP attitudes towards the COVID-19 vaccine and whether beliefs towards other VPDs have changed in the course of the COVID-19 pandemic.

This cross-sectional study aims to assess the knowledge and attitudes of HCP about VPDs before and during the COVID-19 pandemic, and to furthermore estimate the intention of HCP to get vaccinated against COVID-19, and identify determinants that may influence their choice of vaccination through this investigation.

## 2. Materials and Methods

A cross-sectional study for assessing the knowledge and attitudes about VPDs among HCP working at Catania University Hospital (Italy) was conducted between January 2019 to December 2019. The healthcare workforce in this hospital consists of approximately 2800 HCP. The study was performed as part of periodic occupational health surveillance, and is part of a more multifaceted investigation carried out on HCPs approved by the ethics committee of Catania University Hospital (n. 54/2020). Participants were informed about the study aims and procedures and gave their consent to participate.

This study was conducted using a self-administered multiple-choice questionnaire developed by Maltezou et al. [36]. The original version of the questionnaire was translated into Italian by an expert mother-tongue translator. The questionnaire was explained and distributed by trained personnel. The self-administered questionnaires were completed by the HCP anonymously and voluntarily. The following data were collected: demographic and professional issues, knowledge, and attitudes about VPDs.

To proceed with statistical analysis, HCP were grouped according to their tasks: physicians, graduate sanitary (biologists, physicists, chemists, psychologists) (these two groups, according to Italian law, have at least a degree and specialization); nurses and midwives; healthcare assistance staff (physiotherapists, orthoptists, logotherapists); and healthcare diagnostic staff (lab technicians, radio technicians, audiometric technicians, neurophysiopathology technicians). Then, employees were divided by work environment: surgery, medicine, and services. The latter includes support departments, like anesthesiology, radiology, and laboratories.

The COVID-19 pandemic slowed data-processing for this questionnaire. The occupational doctor visits the staff annually; the questionnaire was administered to those seen between September and December 2020. It was not possible to isolate the questionnaires carried out in the previous year and the same period, so they were all processed together. Thus, in September 2020, in the same setting and to HCP who completed the questionnaire in 2019, we administered another questionnaire regarding the intention to get the COVID-19 vaccine. In this circumstance, we re-proposed the old questionnaire.

The survey ended on 20 December 2020. The second questionnaire was structured to have information about: demographic and professional characteristics and attitudes toward mandatory COVID-19 vaccination. Moreover, the HCP’s intention to get vaccinated against COVID-19 was recorded, as well as their reasons for accepting or refusing vaccination.

Data were analyzed by the software SPSS 22.0 (SPSS Inc., Chicago, IL, USA) for Windows. Descriptive analyses were performed using frequencies’ percentages. For the bivariate analysis, the chi-square test was performed to evaluate differences in categorical variables. The level of significance was set at *p* ≤ 0.05. Statistical analyses were conducted using the SPSS, v.22.

## 3. Results

The first questionnaire, conducted in 2019 before the COVID-19 pandemic, was administered to 1354 HCP and 1323 subscribed to the compilation (response rate: 98%). Instead, the second administration was carried out to 798 workers, of whom 787 accepted to participate in the study (response rate: 99%). Features of participating HCP are detailed in Table 1. Most respondents were between the ages of 31 and 50, mirroring the larger workforce in health care facilities in general. Both genders are represented without disparity. Most of the participants were doctors, nurses, and midwifes. The least-represented departments were those of surgery, and in the second administration, 45% of the participants worked in the hospital’s COVID area. Three percent of the interviewed HCPs had contracted laboratory-confirmed COVID.

Table 2 summarizes the HCP’s knowledge regarding the vaccines recommended for HCP by the Italian Vaccination Plan (PNPV 2017-19) before the COVID-19 spread, compared with answers given during the COVID-19 pandemic [37]. HCP had a statistically higher level of correct answers about seasonal influenza, hepatitis B, pertussis, and tetanus-diphtheria vaccines in the pandemic period compared to 2019.

Regarding mandatory vaccination of HCP, the acceptance rates of mandatory vaccination differed by VPD, ranging from 11% for vaccination against hepatitis A, to 64% for vaccination against hepatitis B in 2019, but increased significantly during the COVID-19 pandemic, ranging from 40% for vaccination against hepatitis A, to 82% for the annual influenza vaccination (Table 3). Higher rates of acceptance of mandatory vaccinations for HCP were expressed for HCP caring for immunocompromised patients. HCP favored mandatory vaccinations against all VPDs investigated, except for tetanus-diphtheria in HCP caring for immunocompromised patients compared to all HCP. Acceptance rates increased during the COVID-19 pandemic for all vaccines (Table 3).

When personal circumstances were involved in the investigation, acceptance of mandatory HCW vaccination against VPDs was significantly higher among HCP during the COVID-19 pandemic compared to HCP in 2019 (Table 4).

Table 5 summarizes the intention rates to get a COVID-19 vaccine among HCP according to age, gender, HCP subgroup, educational level, work environment, involvement in COVID-19 care, presence of comorbidity, and the opinion for mandatory and recommendation vaccination against COVID-19. There is a statistically significant difference for gender, profession, and education. Overall, in our study group, 75% of HCP declared that they intended to get vaccinated against COVID-19.

Table 6 shows the reasons to accept or refuse vaccination against COVID-19 among HCP.

## 4. Discussion

This study analyzes new understandings into vaccination knowledge and attitudes before and during the COVID-19 pandemic among a large sample of HCP from one university hospital in Italy. To the best of our knowledge, this is the first study published so far to compare the knowledge and attitudes about vaccines of HCP before and during the COVID-19 pandemic.

The current investigation outcomes showed that the HCP had an overall moderate level of knowledge about all vaccines recommended to them by PNPV 2017–2019 [37]. It is well-recognized that HCP have a central role in vaccinations, and they must have acceptable knowledge to appropriately inform and update the general population, as well as the most fragile and vulnerable patients. Indeed, several recent studies on different target populations in the same area have highlighted that HCP are the most significant and principal sources of information on VPDs in Italy, a country with increased vaccine hesitancy rates among HCP and the general population [38,39,40,41,42]. Consequently, the observed moderate level of knowledge among our study group is to be studied further, and comfort should be found in the improvement that occurred during the COVID-19 pandemic, most probably because of increased compliance with infection control measures in the frame of increased occupational risk perception. However, HCP could undervalue the risks of contracting VPDs and, therefore, of their transmission to other HCP and their patients, principally those in the vulnerable groups. This evidence suggests a necessity for infection prevention and control training.

Instead, HCP play a key role in health-care-associated transmission of VPDs among themselves and patients. Moreover, vaccination of HCP (e.g., against seasonal influenza) may prevent absenteeism and contribute to the preservation of essential healthcare services [43,44,45,46,47,48,49,50,51].

Identification of factors associated with vaccination intentions against VPDs could direct upcoming vaccination campaigns. Regarding mandatory vaccination, there was a moderate acceptance level for HCP who took care of all patients, but it increased significantly for HCP who worked with immunocompromised patients. This also changed favorably during the pandemic period for several occupational vaccinations, namely seasonal influenza, measles, and pertussis. Similar to our findings, HCP favored mandatory vaccination policies for HCP caring for high-risk patients in studies conducted in Greece and Germany [36,52].

Researchers have recognized knowledge as a significant factor inducing HCP’s behavior and attitudes toward vaccination [53,54]. Similarly, HCP with higher levels of knowledge were found to be more receptive to being vaccinated and much more confident in addressing parents’ queries, particularly in settings with time constraints and augmented workloads in their routine medical activity [55].

All HCP must be skilled in occupational exposure risks and preventive and protective procedures. Furthermore, previous studies in the same Italian hospital have shown their low vaccination coverage for VPDs [9,22,23,24,26,28]. This exposes themselves and their patients to VPDs. Consequently, informative campaigns are required to advance the HCP’s familiarity with the recommended vaccinations so as to raise their coverage rates.

Recently, the role of institutions in terms of endorsing mandating vaccination is the subject of ongoing ethics debates. Furthermore, one of the measures recently evaluated was mandating VPDs vaccination among HCP as a requirement for employment [43,56,57,58].

Another result worthy of attention is the response given in personalized scenarios; the vast majority of HCP preferred to have their relatives treated by vaccinated personnel. Similarly, to our findings, Maltezou et al. highlighted the changes in attitudes toward occupational vaccinations in personalized scenarios among HCP employed in various health care settings [36,59,60].

Regarding COVID-19 vaccination, 75% of the respondents in the current study said they would like to be vaccinated. Few studies have been conducted to estimate the intention rate of HCP to get vaccinated against COVID-19. In the Democratic Republic of Congo, only 27.7% of HCP agreed to get a COVID-19 vaccination [61]. An online survey carried out in France reported that 81.5% agreed to get vaccinated [62]. France was faced with a large COVID-19 wave with severe morbidity, hospitalizations, and deaths, similar to Italy. This led to the high declaration of intent for vaccination. Risk perception is strategic for vaccine decision-making.

In our study, the intention rate to get vaccinated was higher in young HCP (≤30 years) and those >51 years of age; to protect themselves and their patients was the main reason reported by HCP who accepted SARS-CoV-2 vaccination. On the other hand, concern about vaccine efficacy and a lack of information about the SARS-CoV-2 vaccine were the main reasons for refusal. Moreover, 65% of HCP reported a perception of not being at risk for infection.

There is also a statistically significant difference for those who are male and those with comorbidities, probably as there is greater awareness of the risk of severe COVID-19 among persons with comorbidities [63]. Men were also more likely to get vaccinated against COVID-19 than women in other studies, and this may be attributed to differences in risk perception between genders [61,64]. The rate of intentions to get vaccinated was also higher among HCP who worked in the COVID area, probably because they could see the severity of the disease and the high mortality rate.

Similarly, in investigations from Congo and Israel [61,64], physicians were more likely to get vaccinated against COVID-19 compared to other HCP. In particular, in our study, 81% of physicians and 70% of nurses and midwives agreed to get vaccinated against COVID-19, which is higher than in nurses from Hong Kong, where only 40% of them stated their willingness to get vaccinated [65]. Similar differences among HCP subgroups regarding vaccinations have been observed in other studies as well, and are attributed to different education levels or professional cultures.

In our study, less than half of participating HCP believed that mandatory vaccination policies against COVID-19 should be implemented for HCP. However, the percentage of HCP that favored compulsory vaccination against COVID-19 was lower than those who thought that vaccinations against VDPs should be mandatory, both before and during the COVID-19 pandemic.

This study highlights some gaps in information and safety concerns that were the main barriers to COVID-19 vaccine acceptance. Gaps in information and misapprehensions about their own risk for contagion, vaccine effectiveness, and safety have already been recognized as barriers to increasing flu vaccine recipients among HCP. Interventions to overcome barriers in getting vaccinated against COVID-19 should be explored to ensure high vaccination rates among HCP.

As with similar studies, it is significant to reflect on some probable limits of this survey. Firstly, the cross-sectional nature limits inferences on the turning connection of the associations detected in this study. Furthermore, the study was carried out in one geographic area of Italy; consequently, the collected responses might not be generalizable to other parts of the country or other countries. However, the fact that the investigation preserved full privacy and anonymity may suggest that the answers were likely to be authentic, with minimal community desirability bias. The high response rate provides an excellent overview of the knowledge and attitudes of HCP. Another limitation is that health care workers may have improved their knowledge during the pandemic because the issue has deepened. In this critical period for public health, so as to successfully address vaccine hesitancy and foster vaccine confidence, evidence-based health communication policies are required.

Finally, it is crucial to recognize the barriers which could lead to anti-COVID-19 vaccine hesitancy early, and urgently implement training plans to be able to reach as many HCP as possible and therefore try to mitigate risks and increase vaccine uptake rates.

In conclusion, the current study shows a gap in knowledge and acceptance of COVID-19 vaccination among HCP; moreover, this investigation reports that the knowledge about recommended vaccinations and acceptance rates of mandatory vaccinations have significantly increased among HCP during the COVID-19 pandemic in Italy. The overall percentage of 75% among the studied HCP that intended to be vaccinated against COVID-19 is considered as good, and may serve as an excellent point to raise vaccine acceptance further after the start of the vaccination campaign in Italy. Additional programs in continuing education about the occupational risk, safety, and usefulness of VPD vaccinations are needed. Consequently, there is a necessity for clear and effective European and national guidelines. Finally, it will be necessary to address the decision of HCPs who do not intend to be vaccinated through clear legislation.

## Figures and Tables

**Table 1:** Sociodemographic and working characteristics of participating HCP (Healthcare personnel) to the survey in the pre-pandemic and ongoing pandemic period.

Variables	Before COVID-19 Pandemic Administration (n. 1323)	During COVID-19 Pandemic Administration (n. 787)
**Age (years)**		
≤30	128 (10%)	77 (10%)
31–40	402 (30%)	241 (31%)
41–50	409 (31%)	268 (34%)
≥51	384 (29%)	201 (25%)
**Male gender**	635 (48%)	368 (47%)
**Health care personnel subgroup**		
Physician	526 (40%)	324 (41%)
Graduate sanitary	38 (3%)	25 (3%)
Nurse and midwife	563 (42%)	357 (46%)
Healthcare assistance staff	108 (8%)	50 (6%)
Healthcare diagnostic staff	88 (7%)	31 (4%)
**Education level**		
Bachelor’s Degree	602 (46%)	364 (46%)
Master’s Degree	157 (12%)	74 (9%)
Post-graduate specialization	506 (38%)	312 (40%)
PhD	58 (4%)	37 (5%)
**Department (or clinic)**		
Surgery	388 (29%)	189 (24%)
Medicine	472 (36%)	296 (38%)
Services	463 (35%)	302 (38%)
**Involvement in COVID-19 care**	NA	354 (45%)
**Personal history of COVID-19**	NA	26 (3%)
**Presence of comorbidities**	unknown	269 (34%)

NA: non-applicable.

**Table 2:** HCP’s knowledge of Italian Vaccination Plan recommended vaccines, the correct response.

Vaccine	Before COVID-19 Administration (n. 1323)	During COVID-19 Administration (n. 787)	*p*-Value
Seasonal influenza annually	854 (65%)	748 (95%)	<0.05
Measles	1201 (91%)	741 (94%)	n.s.
Mumps	1141 (86%)	730 (93%)	n.s.
Rubella	1148 (87%)	754 (96%)	n.s.
Varicella	1194 (90%)	739 (94%)	n.s.
Hepatitis A	676 (51%)	370 (47%)	n.s.
Hepatitis B	1035 (78%)	768 (98%)	<0.05
Pertussis	846 (64%)	691 (88%)	<0.05
Tetanus-diphtheria	697 (53%)	664 (84%)	<0.05

n.s. not significative; HCP: healthcare personnel; COVID-19: coronavirus disease 2019.

**Table 3:** Attitudes towards occupational vaccination for HCP.

Vaccination Should Be Mandatory against	HCP Who Favor Mandatory Vaccinations for All HCP			HCP Who Favor Mandatory Vaccinations for HCP Who Care for Immunocompromised Patients		
	Before COVID-19 Administration (n. 1323)	During COVID-19 Administration (n. 787)	*p*-Value	Before COVID-19 Administration (n. 1323)	During COVID-19 Administration (n. 787)	*p*-Value
Seasonal influenza	748 (57%)	645 (82%)	<0.05	947 (72%)	731 (93%)	<0.05
Measles	478 (36%)	554 (70%)	<0.05	694 (52%)	638 (81%)	<0.05
Mumps	697 (53%)	498 (63%)	n.s.	841 (64%)	603 (77%)	n.s.
Rubella	426 (32%)	524 (67%)	<0.05	748 (57%)	612 (78%)	n.s.
Varicella	559 (42%)	536 (68%)	<0.05	832 (63%)	594 (75%)	n.s.
Hepatitis A	147 (11%)	314 (40%)	<0.05	314 (24%)	416 (53%)	n.s.
Hepatitis B	841 (64%)	637 (81%)	<0.05	965 (73%)	649 (82%)	n.s.
Pertussis	648 (49%)	597 (76%)	<0.05	712 (54%)	603 (77%)	<0.05
Tetanus-diphtheria	584 (44%)	472 (60%)	n.s.	601 (45%)	501 (64%)	n.s.

n.s. not significative; HCP: healthcare personnel.

**Table 4:** HCP’s attitudes regarding mandatory vaccination for HCP using a personalized scenario.

Question	Before COVID-19 Administration (n. 1323)	During COVID-19 Administration (n. 787)	*p*-Value
If a member of your family is immunocompromised, should HCP caring for him be immune against measles?	941 (71%)	698 (89%)	<0.05
If your newborn baby is hospitalized, should HCP in the neonatal intensive care unit be immune against varicella?	1120 (85%)	741 (94%)	<0.05
If a member of your family has chronic obstructive pulmonary disease, should HCP caring for him be vaccinated against influenza?	998 (75%)	713 (91%)	<0.05

HCP: healthcare personnel.

**Table 5:** Intention and attitudes to get vaccinated against COVID-19 by characteristics of HCP.

Variables	Intention to Get Vaccinated against SARS-CoV-2 (n. 787)	*p*-Value
**Age (years)**		
≤30	63 (82%)	n.s.
31–40	186 (77%)	
41–50	174 (65%)	
≥51	170 (85%)	
**Gender**		
Male	312 (85%)	<0.05
Female	225 (54%)	
**Health care workers (HCP) subgroup**		
Physician	261 (81%)	<0.05
Graduate sanitary	12 (48%)	
Nurse and midwife	251 (70%)	
Healthcare assistance staff	42 (84%)	
Healthcare diagnostic staff	27 (87%)	
**Schooling**		
Bachelor’s Degree	223 (61%)	<0.05
Master’s Degree	60 (81%)	
Post-graduate specialization	280 (90%)	
PhD	30 (81%)	
**Work environment**		
Surgery	145 (77%)	<0.05
Medicine	254 (86%)	
Services	194 (64%)	
**Involvement in COVID-19 care**		
Yes	314 (89%)	<0.05
No	279 (64%)	
**Presence of comorbidities**		
Yes	230 (86%)	<0.05
No	363 (70%)	
**COVID-19 vaccination should be mandatory for HCP**		
Yes	341 (43%)	<0.05
No	446 (57%)	
**Intend to recommend SARS-CoV-2 vaccination to high-risk patients**		
Yes	512 (65%)	<0.05
No	275 (35%)	

n.s. not significative; HCP: healthcare personnel; COVID-19: coronavirus disease 2019.

**Table 6:** Reason for accepting or refusing COVID-19 vaccination by HCP in Italy.

Reason	HCP Response(n. 787)
Reason for accepting *	**n. 593**
To protect themselves	484 (82%)
To protect their family	423 (71%)
To protect their patients	469 (79%)
To contribute to the control pf the pandemic	415 (70%)
Reason for refusing *	**n.194**
Concerns about vaccine efficacy	63 (32%)
Concern about vaccine safety	152 (78%)
Perception that COVID-19 is not a dangerous disease	52 (27%)
Not enough information about the vaccine	141 (73%)
Perception of not being at risk for infection	126 (65%)
Other reason	23 (12%)

* More than one answer could be given.

## Data Availability

The data presented in this study are available on request from the corresponding author.

## References

[1] WHO Immunization WHO. http://www.who.int/topics/immunization/en/.

[2] Plotkin S.A., Plotkin S.L. (2011). The development of vaccines: How the past led to the future. Nat. Rev. Microbiol..

[3] Bianchi F.P., Vimercati L., Mansi F., De Nitto S., Stefanizzi P., Rizzo L.A., Fragnelli G.R., Cannone E.S.S., De Maria L., LaRocca A.M.V. (2020). Compliance with immunization and a biological risk assessment of health care workers as part of an occupational health surveillance program: The experience of a university hospital in southern Italy. Am. J. Infect. Control.

[4] Holzmann H., Wiedermann U. (2019). Mandatory vaccination: Suited to enhance vaccination coverage in Europe?. Eurosurveillance.

[5] Maltezou H.C., Ledda C., Rapisarda V. (2019). Mandatory vaccinations for children in Italy: The need for a stable frame. Vaccine.

[6] Scavone C., Sessa M., Clementi E., Rossi F., Capuano A. (2018). Italian Immunization Goals: A Political or Scientific Heated Debate?. Front. Pharmacol..

[7] Sprengholz P., Betsch C. (2020). Herd immunity communication counters detrimental effects of selective vaccination mandates: Ex-perimental evidence. EClinicalMedicine.

[8] Maltezou H.C., Botelho-Nevers E., Brantsæter A.B., Carlsson R.-M., Heininger U., Hübschen J.M., Josefsdottir K.S., Kassianos G., Kyncl J., Ledda C. (2019). Vaccination of healthcare personnel in Europe: Update to current policies. Vaccine.

[9] Ledda C., Rapisarda V., Maltezou H.C., Contrino E., Conforto A., Maida C.M., Tramuto F., Vitale F., Costantino C. (2020). Coverage rates against vaccine-preventable diseases among healthcare workers in Sicily (Italy). Eur. J. Public Health.

[10] MacDonald N.E., Eskola J., Liang X., Chaudhuri M., Dube E., Gellin B., Goldstein S., Larson H., Manzo M.L., Reingold A. (2015). Vaccine hesitancy: Definition, scope and determinants. Vaccine.

[11] Dubé E., Laberge C., Guay M., Bramadat P., Roy R., Bettinger J. (2013). Vaccine hesitancy: An overview. Hum. Vaccines Immunother..

[12] Dubé E., Vivion M., MacDonald N.E. (2015). Vaccine hesitancy, vaccine refusal and the anti-vaccine movement: Influence, impact and implications. Expert Rev. Vaccines.

[13] World Health Organization Ten Threats to Global Health in 2019. https://www.who.int/news-room/spotlight/ten-threats-to-global-health-in-2019.

[14] Calderón T.A., Coffin S.E., Sammons J.S. (2014). Preventing the Spread of Pertussis in Pediatric Healthcare Settings. J. Pediatr. Infect. Dis. Soc..

[15] Apisarnthanarak A., Puthavathana P., Kitphati R., Auewarakul P., Mundy L.M. (2008). Outbreaks of Influenza A Among Nonvaccinated Healthcare Workers: Implications for Resource-Limited Settings. Infect. Control Hosp. Epidemiol..

[16] Lanini S., Ćosić G., Menzo S., Puro V., Durić P., Garbuglia A.R., Milošević V., Karać T., Capobianchi M.R., Ippolito G. (2014). A large epidemic of hepatitis B in serbia: An integrated model for outbreak investigations in healthcare settings. Infect. Control Hosp. Epidemiol..

[17] Yasmin S., Sunenshine R., Bisgard K.M., Wiedeman C., Carrigan A., Sylvester T., Garcia G., Rose K., Wright S., Miller S. (2013). Healthcare-Associated Pertussis Outbreak in Arizona: Challenges and Economic Impact, 2011. J. Pediatr. Infect. Dis. Soc..

[18] Rothan-Tondeur M., de Wazieres B., Lejeune B., Gavazzi G. (2006). Influenza vaccine coverage for healthcare workers in geriatric set-tings in France. Aging Clin. Exp. Res..

[19] Chodick G., Ashkenazi S., Lerman Y. (2006). The risk of hepatitis A infection among healthcare workers: A review of reported outbreaks and sero-epidemiologic studies. J. Hosp. Infect..

[20] Simeonsson K., Summers-Bean C., Connolly A. (2004). Influenza vaccination of healthcare workers: Institutional strategies for im-proving rates. N. C. Med. J..

[21] Steingart K.R., Thomas A.R., Dykewicz C.A., Redd S.C. (1999). Transmission of measles virus in healthcare settings during a communi-tywide outbreak. Infect Control Hosp. Epidemiol..

[22] Ledda C., Cinà D., Garozzo S.F., Vella F., Consoli A., Scialfa V., Proietti L., Nunnari G., Rapisarda V. (2019). Vaccine-preventable disease in healthcare workers in Sicily (Italy): Seroprevalence against measles. Future Microbiol..

[23] Ledda C., Cinà D., Garozzo S.F., Senia P., Consoli A., Marconi A., Scialfa V., Nunnari G., Rapisarda V. (2019). Tuberculosis screening among healthcare workers in Sicily, Italy. Future Microbiol..

[24] Rapisarda V., Nunnari G., Senia P., Vella F., Vitale E., Murabito P., Salerno M., Ledda C. (2019). Hepatitis B vaccination coverage among medical residents from Catania University Hospital, Italy. Future Microbiol..

[25] Celikbas A., Ergonul O., Aksaray S., Tuygun N., Esener H., Tanır G., Eren S., Baykam N., Guvener E., Dokuzoguz B. (2006). Measles, rubella, mumps, and varicella seroprevalence among health care workers in Turkey: Is prevaccination screening cost-effective?. Am. J. Infect. Control.

[26] Costantino C., Ledda C., Genovese C., Contrino E., Vitale E., Maida C.M., Squeri R., Vitale F., Rapisarda V. (2019). Immunization Status against Measles of Health-Care Workers Operating at Three Sicilian University Hospitals: An Observational Study. Vaccines.

[27] Kanamori H., Tokuda K., Ikeda S., Endo S., Ishizawa C., Hirai Y., Takahashi M., Aoyagi T., Hatta M., Gu Y. (2014). Prevaccination Antibody Screening and Immunization Program for Healthcare Personnel against Measles, Mumps, Rubella, and Varicella in a Japanese Tertiary Care Hospital. Tohoku J. Exp. Med..

[28] Costantino C., Ledda C., Squeri R., Restivo V., Casuccio A., Rapisarda V., Graziano G., Alba D., Cimino L., Conforto A. (2020). Attitudes and Perception of Healthcare Workers Concerning Influenza Vac-cination during the 2019/2020 Season: A Survey of Sicilian University Hospitals. Vaccines.

[29] Kumakura S., Onoda K., Hirose M. (2014). Self-reported histories of disease and vaccination against measles, mumps, rubella and varicella in health care personnel in Japan. J. Infect. Chemother..

[30] Bechini A., Chellini M., Pellegrino E., Tiscione E., Lorini C., Bonaccorsi G., Bonanni P., Boccalini S. (2018). Impact of vaccination programs against measles, varicella and meningococcus C in italyand in tuscany and public health policies in the last decades. J. Prev. Med. Hyg..

[31] Fine P., Eames K., Heymann D.L. (2011). Herd immunity: A rough guide. Clin. Infect Dis..

[32] Gualano M.R., Olivero E., Voglino G., Corezzi M., Rossello P., Vicentini C., Bert F., Siliquini R. (2019). Knowledge, attitudes and beliefs towards compulsory vaccination: A systematic review. Hum. Vaccines Immunother..

[33] Rapisarda V., Ledda C., Maltezou H.C. (2019). Vaccination in healthcare workers: Risk assessment, planning, strategy of intervention and legal implications. Future Microbiol..

[34] Talarek E., Chazan M., Winiarska P., Dembiński Ł., Sobierajski T., Banaszkiewicz A. (2020). How attitudes towards vaccination change in the face of an outbreak. Hum. Vaccines Immunother..

[35] European Commission Authorises First Safe and Effective Vaccine against COVID-19. https://ec.europa.eu/commission/presscorner/detail/en/IP_20_2466.

[36] Maltezou H.C., Katerelos P., Poufta S., Pavli A., Maragos A., Theodoridou M. (2013). Attitudes toward mandatory occupational vac-cinations and vaccination coverage against vaccine-preventable diseases of health care workers in primary health care centers. Am. J. Infect. Control.

[37] Ministerodella Salute Piano Nazionale PrevenzioneVaccinale 2017–2019. http://www.salute.gov.it/imgs/C_17_pubblicazioni_2571_allegato.pdfFine.

[38] Bertoldo G., Pesce A., Pepe A., Pelullo C.P., Di Giuseppe G., The Collaborative Working Group (2019). Seasonal influenza: Knowledge, attitude and vaccine uptake among adults with chronic conditions in Italy. PLoS ONE.

[39] D’Alessandro A., Napolitano F., D’Ambrosio A., Angelillo I.F. (2018). Vaccination knowledge and acceptability among pregnant women in Italy. Hum. Vaccines Immunother..

[40] Napolitano F., D’Alessandro A., Angelillo I.F. (2018). Investigating Italian parents’ vaccine hesitancy: A cross-sectional survey. Hum. Vaccines Immunother..

[41] Napolitano F., Napolitano P., Angelillo I.F. (2017). Seasonal influenza vaccination in pregnant women: Knowledge, attitudes, and behaviors in Italy. BMC Infect. Dis..

[42] Pelullo C.P., Della Polla G., Napolitano F., Di Giuseppe G., Angelillo I.F. (2020). Healthcare Workers’ Knowledge, Attitudes, and Practices about Vaccinations: A Cross-Sectional Study in Italy. Vaccines.

[43] Hakim H., Gaur A.H., McCullers J.A. (2011). Motivating factors for high rates of influenza vaccination among healthcare workers. Vaccine.

[44] Carman W.F., Elder A.G., Wallace L.A., McAulay K., Walker A., Murray G.D., Stott D.J. (2000). Effects of influenza vaccination of health-care workers on mortality of elderlypeople in long-term care: A randomisedcontrolled trial. Lancet.

[45] Potter J., Stott D.J., Roberts M.A., Elder A.G., O’Donnell B., Knight P.V., Carman W.F. (1997). Influenza vaccination of health care workers in long-term-care hospitals reduces the mortality of elderlypatients. J. Infect. Dis..

[46] Hayward A.C., Harling R., Wetten S., Johnson A.M., Munro S., Smedley J., Murad S., Watson J.M. (2006). Effectiveness of an influenza vaccine programme for care home staff to preventdeath, morbidity, and health service use amongresidents: Cluster random-isedcontrolled trial. Br. Med. J..

[47] Lemaitre M., Meret T., Rothan-Tondeur M., Belmin J., Lejonc J.-L., Luquel L., Piette F., Salom M., Verny M., Vetel J.-M. (2009). Effect of Influenza Vaccination of Nursing Home Staff on Mortality of Residents: A Cluster-Randomized Trial. J. Am. Geriatr. Soc..

[48] Salgado C.D., Giannetta E.T., Hayden F.G., Farr B.M. (2004). Preventingnosocomial influenza by improving the vaccine acceptance rate of clinicians. Infect. Control Hosp. Epidemiol..

[49] Godinot L.D., Sicsic J., Lachatre M., Bouvet E., Abiteboul D., Rouveix E., Pellissier G., Raude J., Mueller J.E. (2021). Quantifying preferences around vaccination against frequent, mild disease with risk for vulnerable persons: A discrete choice experiment among French hospital health care workers. Vaccine.

[50] Durovic A., Widmer A.F., Dangel M., Ulrich A., Battegay M., Tschudin-Sutter S. (2020). Low rates of influenza vaccination uptake among healthcare workers: Distinguishing barriers between occupational groups. Am. J. Infect. Control.

[51] Gesser-Edelsburg A., Walter N., Green M.S. (2014). Health care workers—Part of the system or part of the public? Ambivalent risk perception in health care workers. Am. J. Infect. Control.

[52] Wicker S., Marckmann G., Poland G.A., Rabenau H.F. (2010). Healthcare Workers’ Perceptions of Mandatory Vaccination: Results of an Anonymous Survey in a German University Hospital. Infect. Control Hosp. Epidemiol..

[53] Riccò M., Cattani S., Casagranda F., Gualerzi G., Signorelli C. (2017). Knowledge, attitudes, beliefs and practices of occupational physi-cians towards vaccinations of health care workers: A cross sectional pilot study in north-eastern italy. Int. J. Occup. Med. Environ. Health.

[54] Loulergue P., Moulin F., Vidal-Trecan G., Absi Z., Demontpion C., Ménager C., Gorodetsky M., Gendrel D., Guillevin L., Launay O. (2009). Knowledge, attitudes and vaccination coverage of healthcare workers regarding occupational vaccinations. Vaccine.

[55] MacDougall D.M., Halperin B.A., MacKinnon-Cameron D., Li L., McNeil S.A., Langley J.M., Halperin S.A. (2015). The challenge of vaccinating adults: Attitudes and beliefs of the canadian public and healthcare providers. BMJ Open.

[56] Prato R., Tafuri S., Fortunato F., Martinelli D. (2010). Vaccination in healthcare workers: An italian perspective. Expert Rev. Vaccines..

[57] Maltezou H.C., Theodoridou K., Ledda C., Rapisarda V., Theodoridou M. (2019). Vaccination of healthcare workers: Is mandatory vaccination needed?. Expert Rev. Vaccines.

[58] Salerno M., Di Mizio G., Montana A., Pomara C. (2019). To be or not to be vaccinated? That is the question among Italian healthcare workers: A medico-legal perspective. Future Microbiol..

[59] Maltezou H.C., Gargalianos P., Nikolaidis P., Katerelos P., Tedoma N., Maltezos E., Lazanas M. (2012). Attitudes towards mandatory vaccination and vaccination coverage against vaccine-preventable diseases among healthcare workers in tertiary-care hos-pitals. J. Infect..

[60] Maltezou H.C., Lourida A., Katragkou A., Grivea I.N., Katerelos P., Wicker S., Syrogiannopoulos G.A., Roilides E., Theodoridou M. (2012). Attitudes Regarding Occupational Vaccines and Vaccination Coverage Against Vaccine-preventable Diseases Among Healthcare Workers Working in Pediatric Departments in Greece. Pediatr. Infect. Dis. J..

[61] KabambaNzaji M., KabambaNgombe L., NgoieMwamba G., BanzaNdala D.B., MbidiMiema J., LuhataLungoyo C. (2020). Ac-ceptability of vaccination against COVID-19 among healthcare workers in the Democratic Republic of the Congo. Pragmat. Obs. Res..

[62] Detoc M., Bruel S., Frappe P., Tardy B., Botelho-Nevers E., Gagneux-Brunon A. (2020). Intention to participate in a COVID-19 vaccine clinical trial and to get vaccinated against COVID-19 in France during the pandemic. Vaccine.

[63] Vella F., Senia P., Ceccarelli M., Vitale E., Maltezou H., Taibi R., Lleshi A., Rullo E.V., Pellicano G.F., Rapisarda V. (2020). Transmission mode associated with coronavirus disease 2019: A review. Eur. Rev. Med. Pharmacol. Sci..

[64] Dror A.A., Eisenbach N., Taiber S., Morozov N.G., Mizrachi M., Zigron A., Srouji S., Sela E. (2020). Vaccine hesitancy: The next challenge in the fight against COVID-19. Eur. J. Epidemiol..

[65] Wang K., Wong E.L.Y., Ho K.F., Cheung A.W.L., Chan E.Y.Y., Yeoh E.K., Wong S.Y.S. (2020). Intention of nurses to accept coronavirus disease 2019 vaccination and change of intention to accept seasonal influenza vaccination during the coronavirus disease 2019 pan-demic: A cross-sectional survey. Vaccine.

